# What has *Royal Society Open Science* achieved in its first few years?

**DOI:** 10.1098/rsos.211838

**Published:** 2021-12-15

**Authors:** Jeremy K. M. Sanders

**Affiliations:** Yusuf Hamied Department of Chemistry, University of Cambridge, Lensfield Road, Cambridge CB2 1EW, UK

**Keywords:** Science, Society and Policy, Royal Society Open Science, Altmetrics, Registered Reports, Open science, Achievements

## Abstract

It has been a pleasure and a privilege to serve as the first Editor-in-Chief of *Royal Society Open Science* for the past 6 years. I step down at the end of December 2021, having completed two 3-year terms, and am taking the opportunity here to reflect on some of the successes and challenges that the journal has experienced and the innovations that we have introduced. When I was first approached back in 2015, the breadth of the journal, covering the whole of science, resonated with my own interests: my research career has ranged across the entire landscape of chemistry, while my leadership roles have embraced all of science, technology and medicine. The open access ethos, the objective refereeing policy that rejects the idea of only publishing what is in fashion, and the opportunities offered by a new venture that could transcend traditional disciplinary boundaries also all appealed to me. Among our successful innovations are Registered Reports, Replication Studies and the new ‘Science, Society and Policy' section. The challenges have included the transition to paid article processing charges (APCs), whether to resist pressure to retract a controversial paper, and bullying of young female authors by established senior males in the same field. I explore all of these below, provide some statistics on the journal's performance, also cover some of the notable papers we have published, and provide some concluding thoughts.

## 1. Registered reports

*Royal Society Open Science* is not the first journal to offer Registered Reports (RRs) but we are reasonably confident in saying it was the first multi-disciplinary journal to do so; this innovation has been ably led by Chris Chambers one of the developers of the format. Briefly, a two-step peer review process allows editors and reviewers to test a hypothesis and proposed methodology and analysis before authors conduct research, with the emphasis being on the quality of the methodology and analysis rather than ‘impact' of results, or on the correctness of the original hypothesis. This approach helps tackle some elements of publication bias and encourages better research design.

Over 170, initial RR submissions have been made since the journal began to offer them, with the majority in psychology but a growing minority in other subjects. More recently, we have joined with a number of other journals to encourage submission of RRs from the ‘Peer Community In' initiative, which it is hoped will expand the reach of the format.

Taken together with the Replication Studies initiative, RRs are a core element of the journal's mission to encourage better and more open science.

## 2. Replications

As with Registered Reports, Replication Studies have a two-stage peer review process: Stage 1 assesses the proposed study design before authors conduct the study, while Stage 2 assesses the results, with publication almost guaranteed if the authors have conducted the study according to their Stage 1 proposals. *Royal Society Open Science* has encouraged (and received) replication studies from launch, albeit infrequently.

Inspired by the so-called ‘Pottery Barn rule' (i.e. you break it; you pay for it), Replications were launched in 2018 for a number of reasons—partly to build on our credentials as an open science innovator; partly to encourage best-practice (by allowing and encouraging replications of content published in the journal and by the Royal Society, as well as a number of publishers); and partly to help tackle the ‘replication crisis' that is well known in some fields and perhaps quietly ignored in others. More than 45 Stage 1 submissions have come via this route, again mostly in psychology.

## 3. Science, society and policy

The opportunity to break out of traditional disciplinary constraints has attracted unusual submissions from the very beginning, but the article in 2019 from Sir David Spiegelhalter and his colleagues on the difficulty of communicating uncertainty about facts, numbers and science stimulated a train of thought that led to the idea of a new section examining the interactions of science with society and policy. The advent of COVID-19 soon afterwards gave added impetus and urgency. We were delighted to attract Nick Pearce, Professor of Public Policy and Director of the Institute for Policy Research (IPR) at the University of Bath, to be the inaugural Subject Editor, and we have assembled a distinguished International Advisory Board and set of Associate Editors prior to the launch in early-2021.

The first few months have seen an overwhelmingly positive response with more than 50 submissions received, and a range of fascinating publications already. Many of these have been COVID-19-related, but a recent Perspective from Sir Michael Marmot examined life expectancy trends and their relationship with UK government policies, and a number of pieces under review are derived from the UK–US Scientific Forum.

## 4. Attracting submissions: new talent and other special collections

Attracting submissions to a new journal is inevitably not trivial, especially when APCs are introduced, as discussed below. Some of our submissions are papers submitted to other Royal Society journals and deemed scientifically sound but they don't meet those journals' selection (and selective) criteria. A successful collaboration with the Royal Society of Chemistry similarly generates papers in chemistry.

However, we have been keen to attract a new generation of researchers, and to that end have been creating special collections by inviting Royal Society University Research Fellows and Dorothy Hodgkin Fellows, with some Newton Fellowships and Sir Henry Dale Fellows, to contribute. This venture was launched with chemistry: invitations in 2017 led to a modest launch event of the published collection in 2018 at the Royal Society with opening remarks from the President, and closing remarks from the Foreign Secretary. Perhaps most pleasing about the collection was the suggestion among several authors that it might lead to new collaborations; we're also delighted that at least three of the original authors have come back and published again in *Royal Society Open Science*.

We have since commissioned similar collections in astronomy, and the broadly ‘molecular' life sciences of biochemistry, cellular and molecular biology, and genetics and genomics, with the Subject Editors leading. The pattern has been set to try to encourage at least one every 12–18 months.

The first special collection was on City Analytics, a fascinating collection of articles bringing together analysis of disparate new types of large-scale data, such as CCTV, social media, city sensors, retail, utility and population censuses to ask big questions about the evolution and nature of city societies. This is a new kind of science, mining vast and often publicly available datasets, generating unexpected insights and recognizing no disciplinary boundaries. We were delighted when the Alan Turing Institute kindly offered to host a launch event for this collection, featuring a number of talks from contributors.

We are currently publishing papers destined for the special collection on catalysis, and around 20 submissions are expected to a cross-disciplinary Artificial Intelligence collection planned for publication next year collection. More such collections are currently being planned.

## 5. Is anybody noticing us? Evidence from Altmetric and citations

In 2015, the journal's first full year of operation, there were 850 000 downloads; by the end of 2020, this had risen to 4.1m, and 2021 is on course to surpass this figure too. We're not driven by Impact Factor (although it does continue to increase, currently over 2.9), but we are interested to measure the journal's impact and influence. Altmetric monitors mentions of all our publications in blogs and social media such as Twitter.^1^ The most prolific tweeter about *Royal Society Open Science* is David Colquhoun FRS who has tweeted/retweeted about us over 700 times, mostly about his own publications, which are also among the most read.

Over 3000 *Royal Society Open Science* publications have a score in Altmetric. The top 5, which illustrate the breadth of the journal's coverage, are:

**Table:** 

paper title	publication year	Altmetric score to date	DOI	views to date
Violent video game engagement is not associated with adolescents' aggressive behaviour: evidence from a registered report	2019	3955	10.1098/rsos.171474	413230
Susceptibility to misinformation about COVID-19 around the world	2020	2337	10.1098/rsos.201199	76357
Goats prefer positive human emotional facial expressions	2018	1794	10.1098/rsos.180491	33289
The natural selection of bad science	2016	1785	10.1098/rsos.160384	169892
A new two-fingered dinosaur sheds light on the radiation of Oviraptorosauria	2020	1625	10.1098/rsos.201184	9137

What is especially pleasing to me is not only does this top 5 demonstrate the immediacy of research published in the journal (with two of the papers published in October of 2020) but also that the highest scoring paper is a Registered Report—some have expressed concerns that this format kills exciting/interesting research, but this evidence (and a record of over 400 000 views for this paper) suggests otherwise.

Our top 10 cited papers, listed below, offer further evidence of the breadth of coverage.

**Table:** 

paper title	publication year	citations to date	DOI
The deep sea is a major sink for microplastic debris	2014	755	10.1098/rsos.140317
An investigation of the false discovery rate and the misinterpretation of *p*-values	2014	370	10.1098/rsos.140216
MiFish, a set of universal PCR primers for metabarcoding environmental DNA from fishes: detection of more than 230 subtropical marine species	2015	344	10.1098/rsos.150088
The natural selection of bad science	2016	250	10.1098/rsos.160384
Bushmeat hunting and extinction risk to the world's mammals	2016	219	10.1098/rsos.160498
Thioflavin T as an amyloid dye: Fibril quantification, optimal concentration and effect on aggregation	2017	207	10.1098/rsos.160696
Determining consistent prognostic biomarkers of overall survival and vascular invasion in hepatocellular carcinoma	2018	152	10.1098/rsos.181006
Improved community detection in weighted bipartite networks	2016	151	10.1098/rsos.140536
Challenges and opportunities associated with waste management in India	2017	149	10.1098/rsos.160764
Baleen boom and bust: A synthesis of mysticete phylogeny, diversity and disparity	2015	144	10.1098/rsos.140434

Among the many other remarkable papers, we have published I highlight ‘The evolution of popular music: USA 1960–2010’ and ‘The advantage of short paper titles’. The latter paper confirms the value of a policy I have followed in my own publications for many years; it has been cited almost 80 times in journals spanning the entire spectrum of science (mostly in journal editorials!). It must be admitted that the correlation between title length and citations is only slight.

## 6. The transition to article processing charges

From launch in 2014 until the end of 2017, *Royal Society Open Science* was free to read and free to publish, the costs being covered from the surpluses generated by other Royal Society journals. However, with the prospect of those journals publishing ever more open access this was not a sustainable model. Reluctantly, therefore a modest APC, initially less than £1000, was introduced in 2018. We have been charging most authors for publication, but there is a well-publicized waiver scheme to avoid disenfranchising resource-limited potential contributors, and this is entirely separate from any consideration of whether to publish a submission. The impact on submission rates has been only modest and there appears not to be a significant geographical dimension to any changes.

## 7. The challenge of controversial papers

As Editor-in-Chief, I have to take a view on the more troublesome manuscripts or matters that arise. In 2018, we published a paper in theoretical physics which had polarized the original referees and which has generated much disagreement in the field. Most experts feel strongly that the underlying mathematics is misconceived and there has been pressure on the journal to retract the paper. But not everyone agrees that the article is completely without merit, and it is possible that in challenging it new insights might emerge. We have published an Expression of Concern which is associated with the original paper so that any new readers are alerted to the controversy, but have decided that in the interest of open debate the paper should stay available for anyone to read. This has not been an easy discussion or decision, but it has now been endorsed by the Royal Society's Publishing Board.

We should remember that in the history of science there are several examples of papers that had scorn poured on them when first published, but which turned out to be correct and to transform their fields. Most papers that look wrong to the majority of readers turn out indeed to be wrong, but a tiny minority are correct and revolutionary. The responsibility of a scientific journal is to do its best but then to leave the final judgement to future generations.

## 8. Unacceptable behaviour

Occasionally, scientific disagreements become more personal. In one case, a female early career researcher received threatening messages and phone calls from senior male competitors who disagreed with the science in the *Royal Society Open Science* paper of which she was the first author. These messages went beyond science: they threatened that the individual's career could be (deliberately) ruined if she continued to be associated with the senior author. When I made clear that such behaviour was unacceptable, the initial response was hostile, but the tone of future exchanges did improve greatly.

In a separate case, a number of promising female post-doctoral researchers indicated to their supervisor that they would leave science because the response to their publications, including one in *Royal Society Open Science*, from some senior men was so unpleasant. When individuals are so harassed or shouted down, or simply ignored, by their ostensible colleagues that they leave active research, the community is poorer for it, and we should be ashamed when this happens.

## 9. Practical challenges

The journey of a manuscript from submission through to publication requires many steps that need in-house editorial staff and infrastructure together with the involvement of external colleagues to act as Subject Editors, Associate Editors and referees. It's a complex and delicate eco-system for every journal, but *Royal Society Open Science* brings its own particular challenges. The differences in reviewing cultures, and even vocabulary, across so many disparate fields adds complexity for the editorial staff. The objective peer review model allows for good but often incremental research which we expect to have archival value, but the research ecosystem prefers ground-breaking research, which contributes to the challenge of engaging reviewers: *Royal Society Open Science* currently needs to invite an average of 7–9 reviewers per paper to reach a decision stage. This is a growing systemic problem for many journals (too many papers written, and the burden falling on too few individuals) and has been greatly exacerbated by COVID-19, but it adds to the challenge of prompt decision-making.

We have also introduced open reviewing, which may discourage some: all referees' reports are now published along with the paper, but only a small proportion of referees have agreed to have their names published.

The systemic situation is unlikely to improve until reviewer activities are better recognized by funders and employers, but the journal's efforts to recognize reviewers in annual lists and also link-ups with services such as Publons are a salve, with the latter now offering ‘transparent’ peer review—publishing of peer review reports for the journal with DOIs, so the reports become formally and easily citable. A number of reviewers and editors have also been provided with letters of thanks by the editorial office indicating when they have assisted—these have been used in promotion, tenure and immigration settings, so it is to be hoped that the journal is helping the community at the individual level in these ways.

The journal currently receives around 2000 submissions annually. While growth in submissions is in general a good thing it adds to the pressure in keeping decision times to a minimum with limited resources. Expansion of the editorial board has been key to tackling the problem, and it remains a work in progress. Identifying suitable individuals to join the board, encouraging them to join and keeping editors engaged is a challenge for the journal – the model of objective and open peer review encourages some to join but puts others off. Expanding the geographical diversity of the Board is a priority and we would welcome readers' suggestions for suitable individuals.

Plagiarism and the very recent proliferation of paper mills that generate fake material to order have further added to the burden of assessment, though fortunately the routine use of similarity checking software has helped somewhat.

Despite the consistently high numbers of submissions and the COVID-19-induced constraints of remote working, the journal has been able to maintain or even reduce decision times in the last 18 months or so and the whole team will be diligent in prioritizing this metric. It has been helpful to expand the number of Subject Editors to 16, covering 13 broad subject disciplinary sections as well as a dedicated Registered Report/Replication Editor. This expansion has helped to build momentum not only in encouraging high-quality submissions from an impressive array of authors and geographies but also encouraging engagement from a talented Associate Editorial Board, which now comprises more than 220 members, and it is continuing to grow.

## 10. Concluding thoughts

I take particular pleasure in publishing the work of very young authors. Our data-analytic colleagues noted that one paper was attracting an unusual number of downloads—it turned out that one of the authors was not only an undergraduate student but also a Thai pop star. We published a single author paper from a Pakistani high-school student, and recently published a paper co-authored by a school student who had prepared interesting materials while on work experience at a research laboratory. We take every opportunity to publicize such work on social media.

A major strength of the journal lies in its editorial board, especially in the sage advice of the Subject Editors. I have enjoyed the spirited discussions in Editorial Board meetings and have learnt much about many fields remote from my own. The journal's workflows have meant I have been more remote from the Associate Editors, but the expertise and enthusiasm for the journal among them is remarkable. I am indebted to all the Editors for their wisdom and support. I'm also hugely grateful to the journal's in-house team; they have extensive experience and knowledge of the journal and publishing in general—from commissioning content to new journal policies—and have provided great support for my unlikely initiatives.

The journal has evolved rapidly, and has perhaps contributed to a quiet revolution in the way that the Royal Society publishes research. While it has grown substantially, it retains a nimbleness that provides opportunities to innovate. I have enjoyed my time as Editor-in-Chief, and take pride in what the team has achieved. As a reader, I look forward to watching the journal develop further in the capable hands of my successor, Dame Wendy Hall.

## Footnotes

1 See https://www.altmetric.com/explorer/outputs?journal_id%5B%5D=5668333c2a83ee0b398b4567.

